# Wisconsin State Dental Society

**Published:** 1870-11

**Authors:** Chas. C. Chittenden


### Proceedings of Societies.

### WISCONSIN STATE DENTAL SOCIETY.

A meeting of the Dentists of the State of Wisconsin, pursuant to a call issued by several of the most prominent operators, was held at Milwaukee, in the office of Dr. Henry Faville, Sept. 28, 1870.

Dr. Edgar Palmer, of La Crosse, was called to the chair, and Dr. Faville was appointed secretary.

On taking the chair Dr. Palmer expressed his thanks for the honor conferred upon him. He said it had long been his desire that the members of the Dental profession in Wisconsin should become united and work together. He urged the necessity of laying aside all personal animosities and professional jealousies, that by united and harmonious effort the standard of Dental education and Dental science in Wisconsin might be elevated to the high position attained by other States, and that the full purposes for which the convention was called might be successfully and speedily accomplished.

On motion of Dr. Brown, of Milwaukee, the chairman appointed a committee of four to prepare and report a constitution and by-laws, viz: Drs. W. D. Brown, D. W. Perkins, C. W. Barnes and J. M. Smith.

On motion, Dr. Palmer, temporary chairman, was added to that committee.

On motion of Dr. Perkins, a committee of five was appointed to nominate officers for a permanent organization, viz: Drs. D. W. Perkins, J. C. Lukes, E. W. Clark, George A. Sherwood and Albert Solliday.

On motion a committee of three was appointed to present subjects for discussion before the meeting while the other committees were preparing their reports. The subjects presented were, 1st. Deciduous Teeth; 2d. Treatment of Exposed Nerves; 3d. Peridontitis. The discussion of these several subjects was opened and continued for about two hours with interest and profit. Dr. Harris was then introduced to the convention and exhibited an improvement in base plates, called "Pyroxyline."

The convention then adjourned till 8 o'clock, P. M.

EVENING SESSION.

At 8 o'clock the convention was called to order by the chairman.

The committee on constitution and by-laws was called and reported a constitution and by-laws which they had drafted. The report was received and the committee discharged.

The constitution was read article by article and unanimously adopted. The by-laws were then taken up and discussed and adopted.

The meeting then adjourned till the following morning at nine o'clock.

THURSDAY MORNING SESSION.

The convention was called to order by Dr. Palmer. Minutes of previous sessions read and approved.

The committee on nominations reported the following names as officers for the ensuing year: President, D. W. Perkins, of Milwaukee; First Vice President, E. A. Clarke, Beloit; Second Vice President, A. Holbrook, Waukesha; Recording Secretary, Chas. C. Chittenden, Madison; Corresponding Secretary, E. Palmer, La Crosse; Treasurer, J. C. Lukes, Racine. The chair appointed Drs. Solliday and Jennings tellers, and the convention proceeded to the election, which resulted in the complete election of the ticket.

The permanent officers were then installed and the organization of the Wisconsin State Dental Society was declared effected.

On motion of Dr. Palmer, any Dentists present who have had eight years successful practice were admitted to membership upon payment of five dollars and by signing the constitution. The society then adjourned to 2 o'clock P. M.

AFTERNOON SESSION.

The society met pursuant to adjournment, the president in the chair.

The following resolution was introduced by Dr. Holbrook, and unanimously adopted:

*Resolved*, That the thanks of this society are hereby tendered to Dr. Henry Faville for the use of his office for the meetings of this society.

A bill of Dr. Palmer's, for printing circulars, postage, etc., in calling the convention together, amounting to $5.40, was presented and allowed.

The president then announced the following standing committees for the ensuing year:

Executive committee—Drs. E. Palmer, Solliday and Foster.

Publication—Drs. C. C. Chittenden, Goodman and Sherwood.

Membership—Drs. Barnes, Smith and Holbrook.

Dental Ethics—Drs. Brown, Stevens and Washburn.

The following resolutions were introduced by Dr. Palmer and adopted:

*Resolved*, 1. That the president appoint a committee of three to obtain, if possible, from the legislature of this State, at its coming session, a charter of incorporation for the benefit of this society, after form of "bill" accompanying this resolution, or after such other form as may be adopted by the committee.

*Resolved*, 2. That all necessary expenses of procuring the passage of such "bill" be borne by this society.

The president appointed Drs. Palmer, Chittenden and Barnes.

The second resolution of Dr. Palmer, on legislation, was as follows:

*Resolved,* That a committee of three be appointed by the president of this society, whose duty it shall be to have printed a suitable number of blanks, after the form of petition accompanying this resolution, or in such other form as in their judgment shall best secure the object sought, and to place a copy in the hands of all members of this society who will consent to make an effort to obtain signatures thereto, and to use all honorable methods to awaken an interest in the public mind, so as to secure the largest possible number of signatures to the petition, and to report to this society at its next regular meeting.

Further, it shall be the duty of this committee to draft a bill for presentation to the General Assembly of this State for the regulation of the practice of Dentistry in accordance with accompanying petition, and to present the same at the next regular meeting of this society for its consideration and action.

*Resolved,* 2. That the expense of such printing and distribution be borne by this society.

The President appointed the same committe as on charter.

The following resolution was introduced by Dr. A. Holbrook and unanimously adopted:

*Resolved,* That the thanks of this society are due and are hereby heartily and sincerely tendered to Dr. Edgar Palmer for his untiring efforts and indefatigable zeal in calling together the convention, which resulted in the formation of this society.

On motion, the recording and corresponding secretaries were empowered to incur the necessary expense required in procuring such books, blanks, etc., as they shall need in properly conducting the duties of their respective offices.

The society then adjourned to meet in Madison on the second Tuesday in January, 1871.

CHAS. C. CHITTENDEN, *Rec. Sec'y.*

